# How university teachers’ digital literacy influences their innovative ability: a system dynamics theoretical modeling and simulation study

**DOI:** 10.3389/fpsyg.2025.1665337

**Published:** 2026-02-09

**Authors:** Zhangwei Mao, Simiao Tong, Chao Jiang, Sijia Yan, Yuntao Bai

**Affiliations:** 1 Zhejiang University of Finance and Economics Dongfang College, Haining, Zhejiang, China; 2 Department of Business English, Chongqing University of Education, Chongqing, China; 3 Business School, Shandong Management University, Jinan, China

**Keywords:** university teachers, digital literacy, innovation ability, system dynamics, psychological capital

## Abstract

The global digital transformation is driving profound changes in modern education, with university teachers’ digital literacy and innovation capabilities gradually becoming key factors in advancing digital education reform and enhancing a nation’s international competitiveness and soft power. Adopting a dynamic perspective, this study abstractly constructs a system dynamics model spanning from the digital ecosystem layer to the psychological capital layer and ultimately to the innovation output layer. By incorporating simulation data, the study integrates teachers’ digital literacy, psychological factors, and innovation capabilities into the simulation system to examine the impact of digital literacy on teachers’ innovation capacity and reveal its underlying mechanisms. The findings indicate that university teachers’ digital literacy strengthens along the dynamic pathway of “psychological capital accumulation—self-efficacy enhancement—well-being improvement—cognitive closure reduction.” Psychological factors such as teachers’ self-efficacy, flow experience intensity, and well-being play significant mediating roles, while institutional technical support further reinforces this pathway. Notably, when digital technology complexity reaches extremely high levels, teachers’ flow experience intensity drops to its lowest point around the 0.5-period mark, exhibiting a “U”-shaped curve. On one hand, this study addresses gaps in existing research by analyzing the dynamic mechanisms through which teachers’ digital literacy influences innovation capabilities, overcoming the limitations of traditional static theories. On the other hand, it strengthens the digital knowledge framework for teachers and deepens the practical application of dynamic systems theory in educational transformation.

## 1 Introduction

The modern digitalization process has made the educational environment increasingly complex (Hatlevik and Christophersen, 2013). The application and popularization of digital intelligent technologies in the educational environment have gradually become the core part of school daily activities. Their deep penetration into the education system has led to changes in the teaching environment, teaching models, and teaching activities, promoting the systematic transformation and reconstruction of the educational ecosystem. Digital literacy has gradually become an important factor for social and economic development and labor employment and is a key ability for becoming a high-level talent in digital society (Bejaković and Mrnjavac, 2020). The digital literacy of the entire population has gradually become a key indicator for countries to enhance their international competitiveness and soft power. The digital transformation of education has been elevated to the national strategic level by many countries. Among them, teachers, as the first resource of education, play an important driving role in promoting the digital transformation of education. The digital literacy of university teachers has also become a topic of widespread concern in the international community and has been included in national strategic content, becoming an important strategic decision for regions and international organizations around the world (as shown in Table 1).

**Table 1:** Policy documents on the cultivation of teachers’ digital literacy in recent years from a global perspective.

United Kingdom	United States	China	European Union	UNESCO	Developing Countries
2012: Government Digital Strategy	2016: Future ready learning: reimagining the role of technology in education	2018: Opinions on comprehensively deepening the reform of teacher team building in the new era; notice of the Ministry of Education on carrying out pilot work for the action of promoting teacher team building with the help of artificial intelligence	2017: European framework for the digital competence of educators	Reimagining our future together: a new social contract for education	Mexico:@prende 2.0 Platform (2016–2018)
2014: Make or break: the UK’ digital future (Bejaković and Mrnjavac, 2020)	2017: ISTE standards for educators	2019: China education modernization 2035	Erasmus+ Programme (2021–2027)	Artificial intelligence in education: challenges and opportunities for sustainable development	Uganda: Digital education agenda (2022–2026)
2018: AI in the UK: ready, willing and able	2017: Reimagining the role of technology in education: 2017 National education technology plan update	2021: Notice of the Ministry of Education on implementing the second batch of pilot projects for promoting teachers’ team building through artificial intelligence	Digital education action plan (2021–2027)	2017: the UNESCO ICT competency standard for teachers (ICT CST)	Brazil: National digital education policy (Política Nacional de Educação Digital - Pned)
2022: the UK digital strategy	2019: The national artificial intelligence research and development strategic plan	2022: Notice of the Ministry of Education and other eight departments on issuing the ‘strong teacher plan for basic education in the new era’ and “Digital literacy of teachers”		2018: Digital literacy global framework	Africa: Regional teachers initiative for Africa (RTIA) (2024–2027)

In recent years, the UK government has continuously advanced digital education, particularly emphasizing the enhancement of digital literacy among university teachers to accelerate digital economic growth and support the nation’s digital transformation. To this end, it has issued reports such as the “Digital Future Report,” the “UK AI Development Report,” and the “UK Digital Strategy.” These initiatives aim to promote digital education through a series of policies, with improving teachers’ digital literacy as the top priority in cultivating talent for the digital economy. UNESCO has similarly highlighted in documents such as “Artificial Intelligence in Education: Challenges and Opportunities for Sustainable Development” that the integration of artificial intelligence in education represents a crucial trend in the future transformation of education, which will further drive the evolution and restructuring of educational systems. The application of “big data” has also instigated substantial changes in the educational sector, fostering a major transformation of the educational environment. Globally, countries are employing educational data mining and learning analytics technologies to construct relevant models, explore correlations among educational variables, and allocate substantial human and material resources to support the application of big data. Prestigious institutions such as Yale University and Stanford University have initiated research programs focused on educational big data. In December 2019, the outbreak of the coronavirus (COVID-19) led to strict containment measures, including lockdowns and social distancing, forcing educational institutions worldwide to close and abruptly shift to Emergency Remote Education (ERE) (Bozkurt and Sharma, 2022; Bozkurt et al., 2022; Hodges and Fowler, 2020), resulting in an explosive growth of remote learning. This crisis, the first of its kind in the digital knowledge era, has triggered widespread socio-cultural, economic, and political repercussions, with the education sector feeling the impact like the flutter of a butterfly’s wings (Bozkurt and Sharma, 2022). Simultaneously, this transformation has been dubbed the “Great Online Learning Experiment,” revealing what works and what does not, serving as a wake-up call for global education. Educational models must be optimized, with face-to-face and remote education moving toward hybridization and collaboration, requiring more effective and flexible use of educational technology to advance the field further (Bozkurt and Sharma, 2022). All these changes in traditional education models have forced teachers to rethink how to improve their digital literacy to better implement teaching and provide higher-quality teaching content. Digital pedagogy is the art of computer-driven digital technologies that significantly enrich learning, teaching, assessment, and the entire curriculum system (Bećirović, 2023). The rapid evolution and wide application of digital technologies in the education field urgently require teachers to take on new responsibilities and missions. The professional development of teachers is at the center of all school improvement plans (Haleem et al., 2022; Adey, 2004), is the core driving force of educational change, and is also the key to the success or failure of educational change. Educators must enhance their digital literacy and strengthen their ability to apply digital technologies to effectively navigate the digital transformation occurring within the contemporary educational landscape (Kivunja, 2013).

Existing research supports this view. The frontier research on digital literacy has recognized the importance and timeliness of digital literacy, elevating technical knowledge to the core position in the teacher’s knowledge structure (Mishra and Koehler, 2006; Albakri and Wood-Harper, 2025). Research based on resource conservation theory and innovative self-efficacy also demonstrates that adequate initial resources and a positive psychological state encourage individuals to exhibit positive innovative behaviors (Bono et al., 2013; Halbesleben and Wheeler, 2008; Hobfoll et al., 2018). Teachers should be at the forefront of applying artificial intelligence in education (Wang et al., 2023). With the emergence of generative AI like ChatGPT, the integration of AI in education has become a powerful transformative force sweeping through the educational landscape, necessitating a reevaluation of the relationship between technology and pedagogy (Mishra et al., 2023; Ning et al., 2024; Garzón et al., 2025). AI in education helps bridge educational gaps, contributing to the global goal of educational equity (Al-Shahrani, 2024), and teachers must lead the charge in AI-driven education. Damanik and Widodo (2024) found that teachers with high digital literacy are better equipped to integrate information technology, subject matter, and teaching methods, demonstrating stronger professional performance and teaching quality. Psychological resilience plays a crucial mediating role in transforming teachers’ digital literacy into creativity. Teachers with high resilience exhibit greater adaptability in the face of external pressures, technological challenges, and uncertainties in teaching scenarios, enabling more effective conversion of digital literacy into digital teaching capabilities amid technological iterations and institutional reforms (Feng and Sumettikoon, 2024). However, current academic research on the mechanism of how university teachers’ digital literacy influences their innovative capabilities remains insufficient, predominantly limited to theoretical discussions with a lack of quantitative studies. Countries also face challenges such as inadequate institutional frameworks, superficial application of technology deviating from educational goals, and the inertia of traditional educational concepts when it comes to enhancing teachers’ digital literacy and innovative capabilities. Specifically focusing on the impact mechanism of university teachers’ digital elements on their own innovative abilities, existing research is scarce and mostly centers on static empirical analyses, lacking a dynamic perspective. Furthermore, few studies adopt a systems theory approach, integrating technological and psychological factors to holistically analyze how the enhancement of university teachers’ digital literacy systematically affects their innovative capabilities and reveals the underlying mechanisms. Therefore, this paper conducts theoretical modeling and simulation research based on system dynamics. Drawing on the Conservation of Resources Theory, it constructs a system dynamics model spanning the digital ecological layer, psychological capital layer, and innovation output layer. Using system dynamics methods, it quantifies factors such as university teachers’ digital elements and innovative self-efficacy to explore the impact mechanism of their digital literacy on their innovative capabilities. Following the dominant logic of “university teachers’ digital literacy—various influencing factors (black box)—innovative capabilities,” this study aims to investigate the “black box problem” by addressing the following fundamental questions: First, whether university teachers’ digital literacy can influence their innovative capabilities; second, the roles of flow experience, teachers’ self-efficacy, and teachers’ well-being in the process of digital literacy affecting innovative capabilities; and third, the pathways through which teachers’ digital literacy influences their innovative capabilities.

This study investigates the interplay between the technical and psychological dimensions by constructing a system dynamics model to explore the relationship between university teachers’ digital literacy and their innovation capabilities. Using the system dynamics approach, this research aims to describe and quantify the influence of technical and psychological factors, simulating the impact of digital literacy on teachers’ innovation capabilities and identifying the underlying mechanisms. On one hand, system dynamics provides a powerful tool for research, helping to construct a dynamic model of the interaction between digital literacy and innovation capability among university teachers. It more clearly reveals the causal relationships and feedback mechanisms, addressing the theoretical gap in understanding the dynamic mechanisms of enhancing teachers’ innovation capacity, while offering a more reliable methodological reference for subsequent studies. On the other hand, through simulation research, different policy interventions or solutions can be modeled to obtain predictive data, providing a more scientific theoretical basis for decision-making applications by universities and educational management authorities. Furthermore, by incorporating psychological factors such as “psychological capital,” the study explores the psychological motivations driving the enhancement of teachers’ innovation capabilities, helping to address the practical issue of insufficient developmental momentum in traditional teacher-centered approaches. This research can also be regarded as a new exploration of second-order SD models in the field of educational management. It strengthens teachers’ digital knowledge systems, improves their digital application abilities, and deepens the application of dynamic systems theory in educational transformation.

## 2 Literature review and methods

### 2.1 Key variable connotations

In the 1990s, Gilster (1997) defined “digital literacy” in his book “Digital literacy” as “the ability to access, understand, and use information on the internet.” Over time, the concept of digital literacy has been progressively enriched. It has evolved from an initial focus on the general skills required to master digital technology applications to a broader emphasis on the comprehensive qualities, including knowledge, skills, and attitudes, necessary for the judicious and innovative use of digital technology. Teacher digital literacy is an important extension and development of digital literacy in the field of education. Teacher digital literacy has gradually occupied a core position in the knowledge structure of teachers (Mishra and Koehler, 2006). From the perspective of concept connotation, Krumsvik suggested that teacher digital literacy is the proficiency of teachers in using ICT (information and communication technology) in professional environments. Research indicates that the cultivation of digital literacy can be facilitated through digital education technologies, thereby promoting the development of teaching competencies (Gabriel F. et al., 2022). Preservice teachers’ perception of digital literacy can directly and positively influence their ICT self-efficacy, contributing to improved teaching experiences (Gao et al., 2025). Possessing digital literacy is a fundamental requirement for contemporary teachers, and teachers’ digital literacy can be influenced by generational differences (Oktavia, 2024). In this study, teacher digital literacy is defined as an individual’s interest, attitude, and ability to appropriately use digital and communication technologies to access, manage, integrate, and evaluate information, construct new knowledge, and communicate with others to participate effectively in society (Tejedor et al., 2020). Based on the “gain principle” of the Conservation of Resources Theory and Social Cognitive Theory, teacher digital literacy is regarded as a cumulative resource in teachers’ professional lives. This “resource” can be transformed into teachers’ innovative behaviors, fostering the enhancement of their innovative capabilities (Gkontelos et al., 2023).

Flow is a subjective state reported by people when they are fully engaged in something to the extent that they forget time, fatigue, and everything else except the activity itself. Its defining feature is a strong experience of participation in every moment of the activity, with complete attention focused on the task at hand. In this state, the person can fully utilize his or her abilities (Csikszentmihalyi, 1988). Flow has an impact on the intrinsic motivation of human behavior. Most of the rewards of intrinsically motivated behavior come from the experience of absorption and interest, and the epitome of this is flow (Csikszentmihalyi, 1988). The flow experience is an expanding force related to an individual’s goal and interest structure, as well as a force for skill growth related to existing interests (Csikszentmihalyi and Larson, 2014). The intensity of flow experience in this study refers to what Csikszentmihalyi described, specifically highlighting teachers’ strong sense of engagement when integrating digital technology into the classroom, along with an intrinsic motivational feedback driving them to continuously and voluntarily incorporate digital technology into teaching.

Self-efficacy refers to an individual’s belief in their ability to perform specific tasks or achieve certain goals (Denzin et al., 2006; Heng and Chu, 2023; Bandura, 2000a, Bandura, 1991, Bandura, 2000b, Otmane et al., 2020). This belief can influence life events and is a core element of Bandura’s social cognitive theory (Pajares, 2002; Perkmen and Pamuk, 2011; Van Dinther et al., 2011). Teachers’ self-efficacy, like general self-efficacy beliefs, reflects their confidence in completing specific tasks (Bandura, 2000a; Bandura, 2018). Teachers’ self-efficacy directly manifests in daily teaching, affecting instructional methods and classroom management (Kass, 2013; Gkontelos et al., 2023). Self-efficacy is a significant positive predictor of innovative teaching practices, playing a crucial role in promoting innovative pedagogy (Li et al., 2025). This study primarily focuses on teachers’ innovative self-efficacy and technological self-efficacy. Innovative Self-efficacy is an intrinsic cognitive state, representing an individual’s confidence in successfully innovating, and serves as a vital psychological resource. Technological self-efficacy denotes an individual’s belief in their ability to use information technology to accomplish specific tasks (Ramazani and Talebi, 2023), emphasizing teachers’ capacity to integrate digital tools such as Web 2.0 technologies and software applications into classrooms and curricula. It highlights confidence in using technology, which is both task-specific and task-dependent (Albion, 1999; Artino, 2012; Bandura, 2000a; Holden and Rada, 2011).

Well-being is based on satisfaction with key areas of life and, as one of the essential components of PsyCap, has been proven to predict satisfaction with work, health, relationships, and life in general (Luthans et al., 2013), including the educational domain. In this study, teacher well-being specifically refers to university instructors’ satisfaction with teaching using technology and integrating digital tools into the classroom.

In this study, innovation ability refers to the capacity of teachers to proactively engage in innovative behaviors. Innovative behavior is defined as the process of first identifying a problem, then generating new solutions, seeking support, and ultimately transforming them into objective actions (Scott and Bruce, 1994). Janssen (2004) further expanded innovative behavior into four dimensions: idea generation, idea implementation, idea promotion, and idea diffusion. Innovative behavior is a role-extraneous behavior, often manifested as flexible or even rule-breaking prosocial deviance (Eschenbacher and Fleming, 2020), which is generally not covered by organizational rewards and requires individuals to invest more resources proactively. Teachers’ innovative behavior refers to the process of proposing and implementing new ideas and technologies in the teaching process.

### 2.2 Theoretical review of key variable relationships

**Figure 1:**
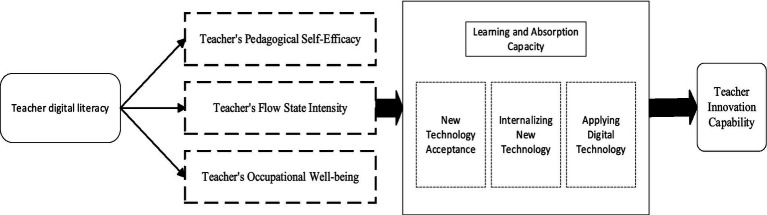
Key conceptual model of the relationship between teachers’ digital literacy, psychological factors, and innovative capacity.

From the perspective of the “resource gain principle” in Conservation of Resources Theory and Social Cognitive Theory, the enhancement of teachers’ digital literacy and the leap in their innovative capabilities are essentially a process of continuous resource accumulation and transformation. This study proposes a key conceptual model of the relationships among teachers’ digital literacy, psychological factors, and their innovative capabilities. The leap in teachers’ innovative abilities is influenced by the chain transmission effect of psychological variables: the increase in teachers’ self-efficacy, the intensity of their flow experiences, and their sense of well-being stimulate their absorption of new technologies and improvement in learning abilities. By accepting, internalizing, and applying new technologies, teachers ultimately enhance their innovative capabilities. This process represents the transformative activity through which teachers’ digital literacy affects the leap in their innovative capabilities. Additionally, this study later extends the model by incorporating external factors such as school support and technological complexity, as well as inhibitory variables like digital fatigue, using a second-order SD model to explore the mechanisms by which teachers’ digital literacy influences their innovative capabilities (Figure 1).

### 2.3 A brief feedback loop between digital literacy and innovation ability

#### 2.3.1 The “digital literacy–flow experience cycle” brief feedback loop

**Figure 2:**
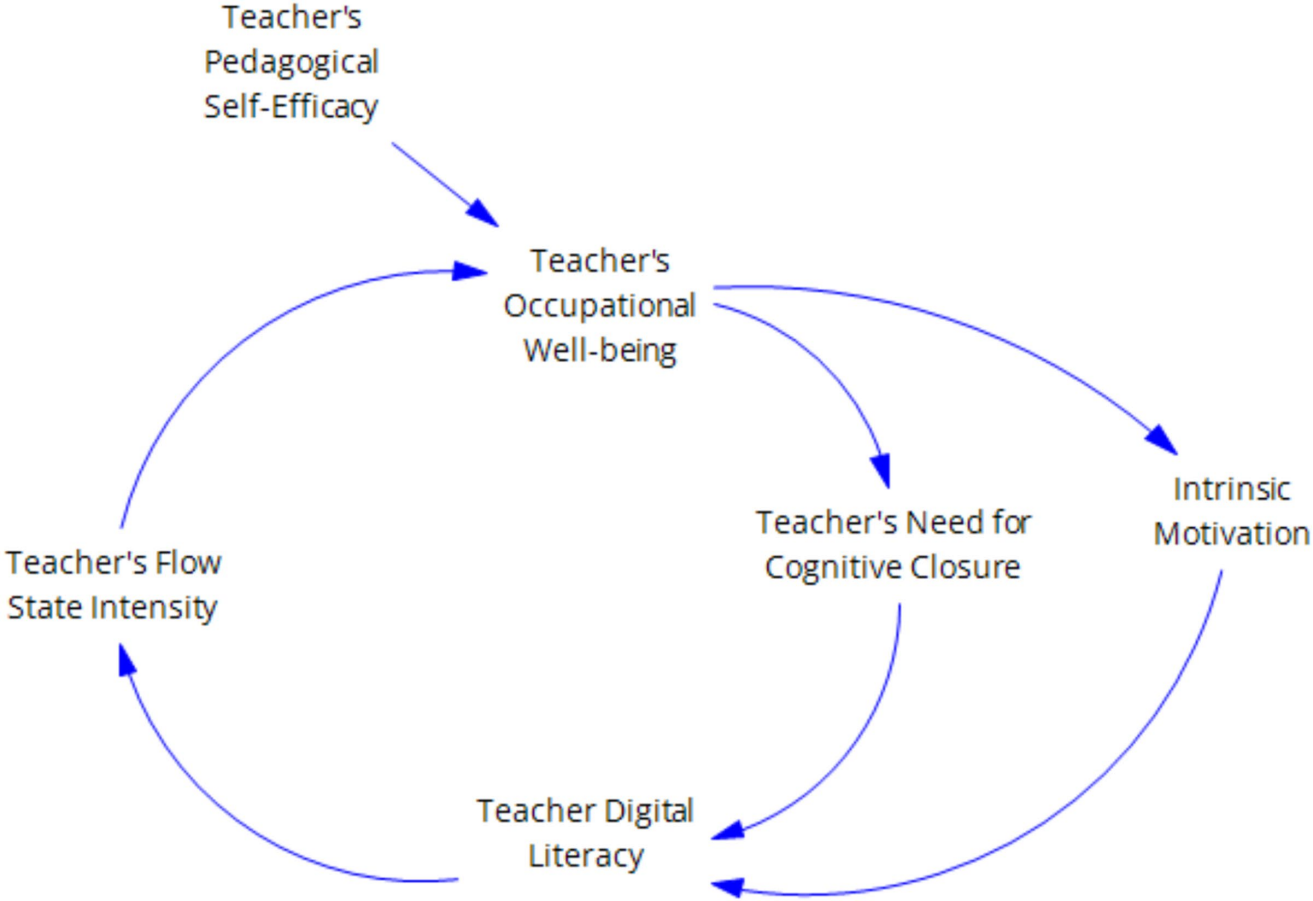
Demonstrates the reinforcing cycle of “digital literacy—flow experience”.

**Figure 3:**
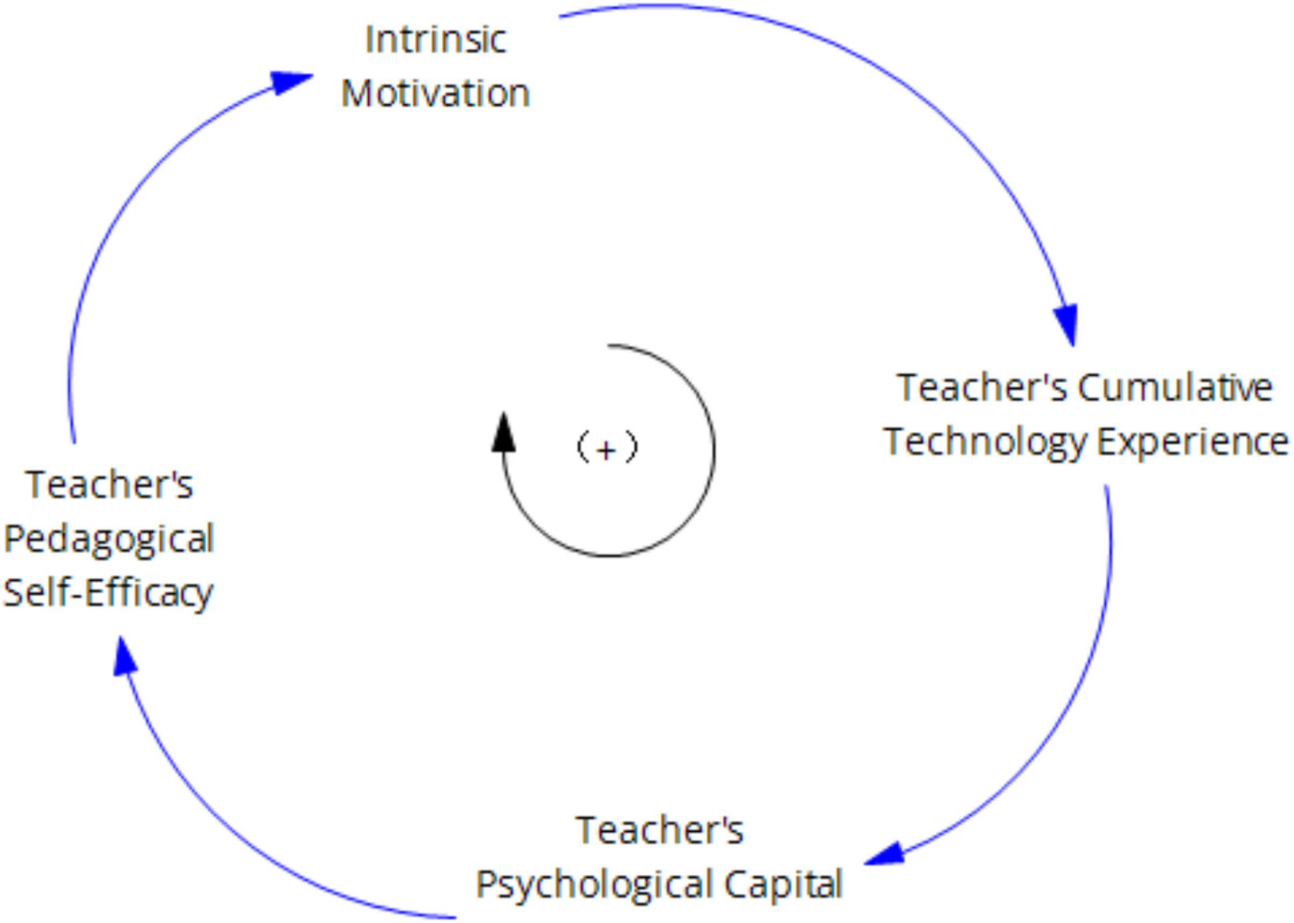
Illustrates the reinforcing cycle of “Teachers’ Psychological Capital-Self-Efficacy”.

**Figure 4:**
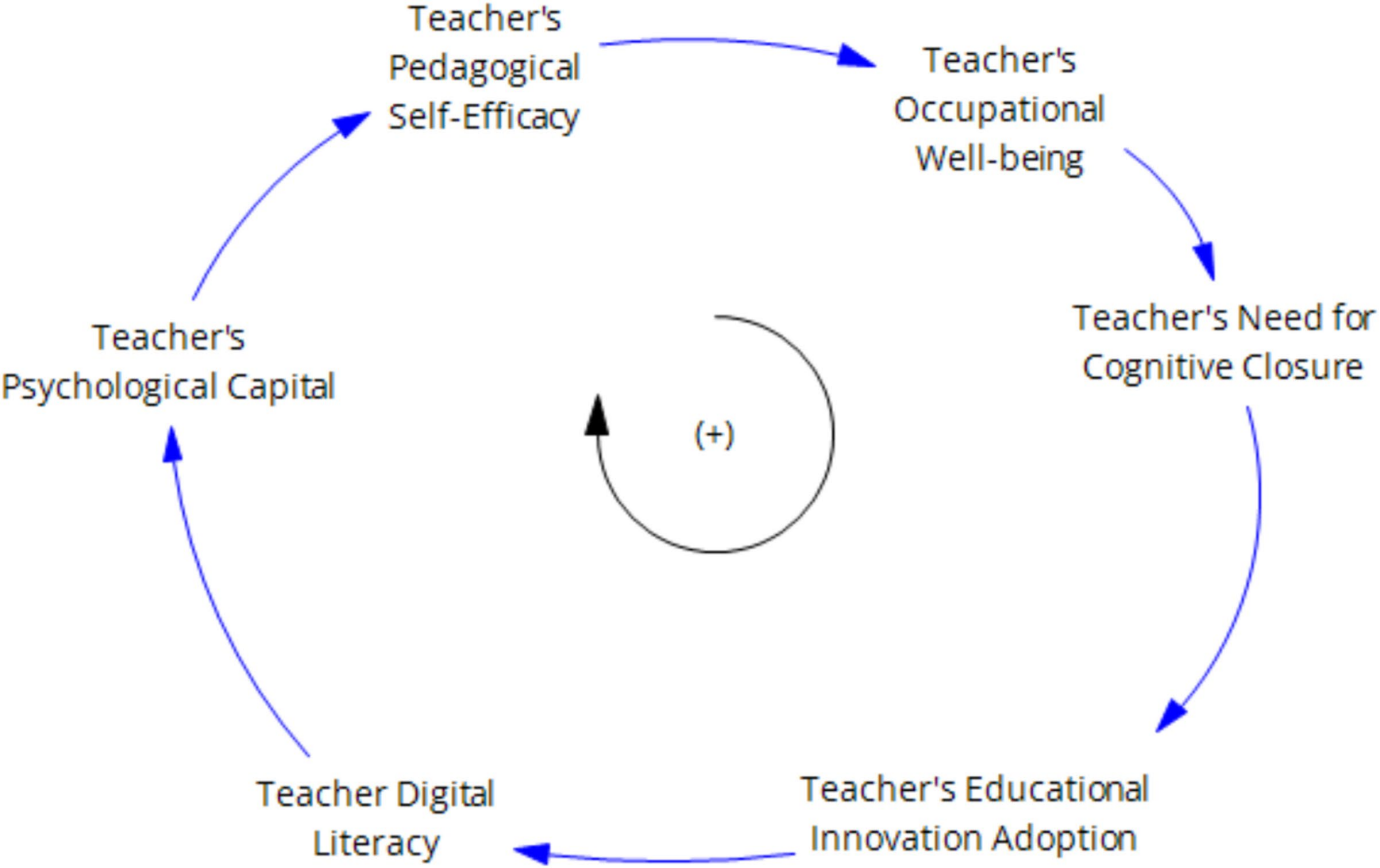
“Digital literacy—psychological variables—innovation behavior level” reinforcement loop.

Research based on the conservation of resources theory and innovative self-efficacy has also demonstrated that sufficient initial resources and positive psychological states promote individuals’ display of positive innovative behaviors (Bono et al., 2013; Hobfoll et al., 2018) As an initial resource, teachers’ digital literacy, along with psychological resources such as self-efficacy and teacher well-being, under the basic fact that individuals with abundant initial resources have stronger abilities to acquire resources and can exhibit more positive mindsets and work behaviors, we can conclude that enhancing teachers’ digital literacy, strengthening their self-efficacy, and thereby intensifying their flow experience can promote the improvement of teachers’ innovation capabilities. As shown in Figures 2–4.

#### 2.3.2 “Teacher wellbeing—cognitive lock-in” brief feedback loop

**Figure 5:**
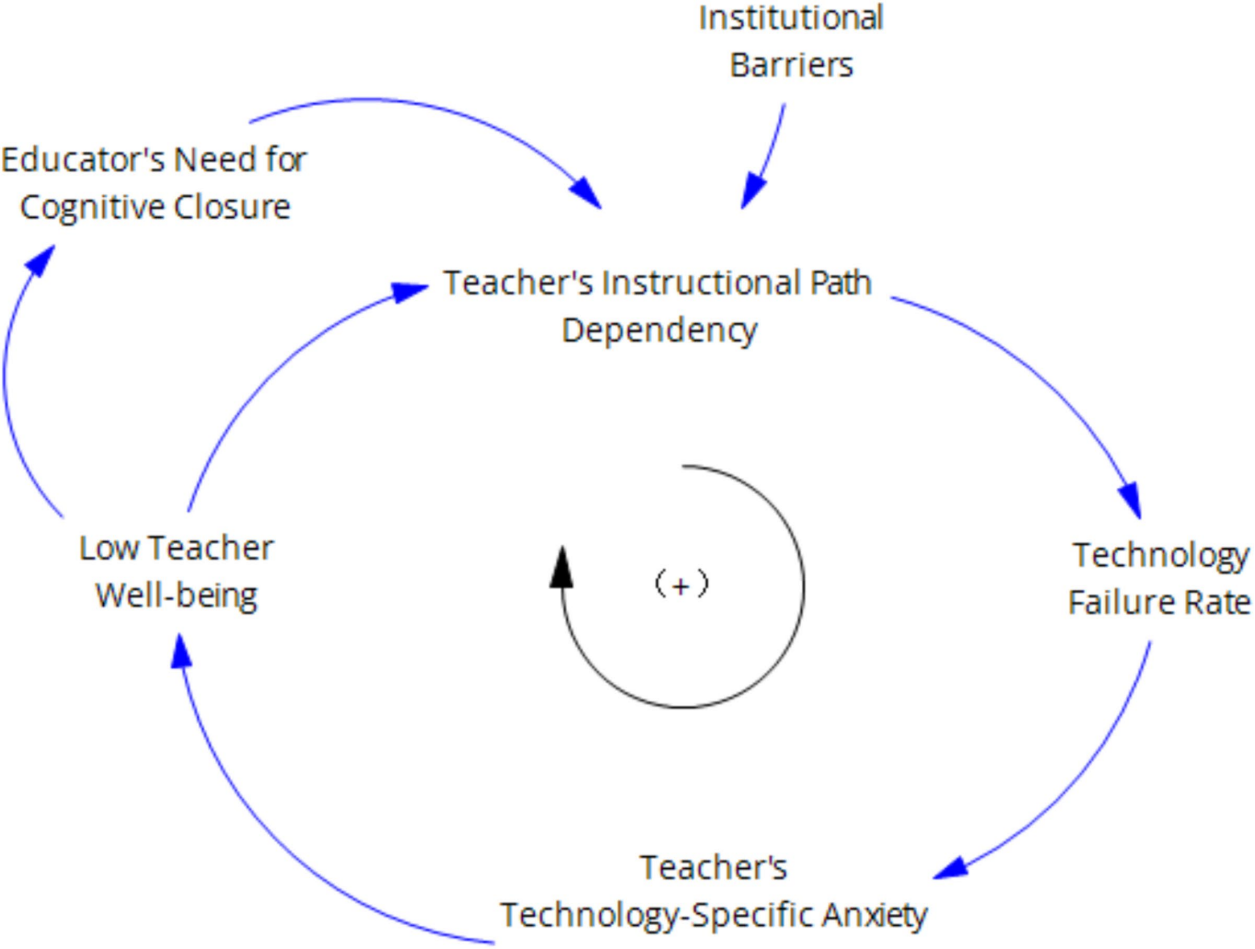
Negative teacher well-being inhibition feedback loop.

First, studies based on social cognitive theory and self-determination theory both agree that negative psychological factors (such as well-being and self-efficacy) can prompt individuals to exhibit negative mindsets and work behaviors (Gabriel, A. S. et al., 2022), leading to a “cognitive lock-in effect” that reduces the occurrence of individual innovative behaviors and thereby weakens innovation capabilities. Teacher technological anxiety stimulates a decrease in teachers’ technological well-being, which in turn enhances their need for cognitive closure, strengthens their reliance on traditional teaching paths, and further intensifies their technological anxiety. Ultimately, the occurrence of teachers’ innovative behaviors decreases, weakening their level of innovative behavior. As shown in Figure 5.

#### 2.3.3 A brief feedback loop of “teacher digital literacy—digital avoidance—innovative behavior”

**Figure 6:**
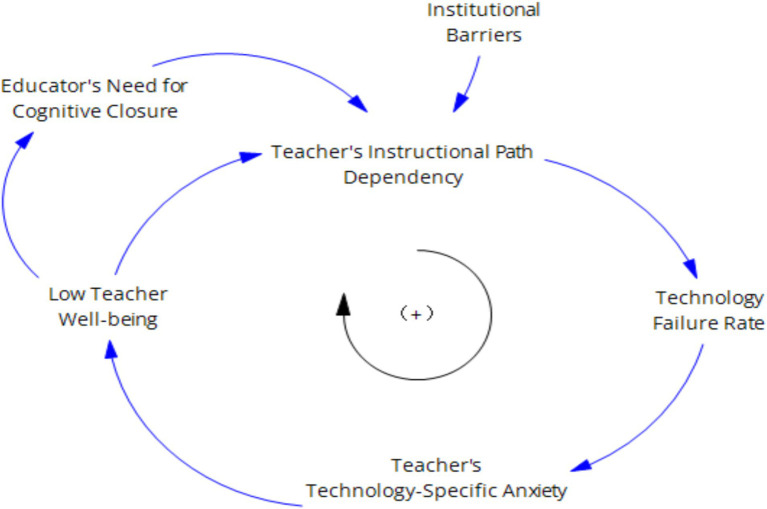
“Teacher digital literacy—digital avoidance—innovative behavior” inhibitory feedback loop.

The cultivation of digital literacy can promote the development of teaching abilities (Gabriel F. et al., 2022). The degradation of teachers’ digital literacy increases the occurrence rate of technical malfunctions, intensifies self-doubt in technology use, and leads to an increase in digital avoidance behavior among teachers, which in turn enhances the forgetting of innovative behavior and weakens the level of teachers’ innovative behavior. The weakening of teachers’ innovative behavior further exacerbates the rate of digital literacy degradation, forming a vicious cycle. As shown in Figure 6.

#### 2.3.4 “Teacher digital fatigue—digital avoidance—decline in innovative behavior—degradation of digital literacy” simplified feedback loop

**Figure 7:**
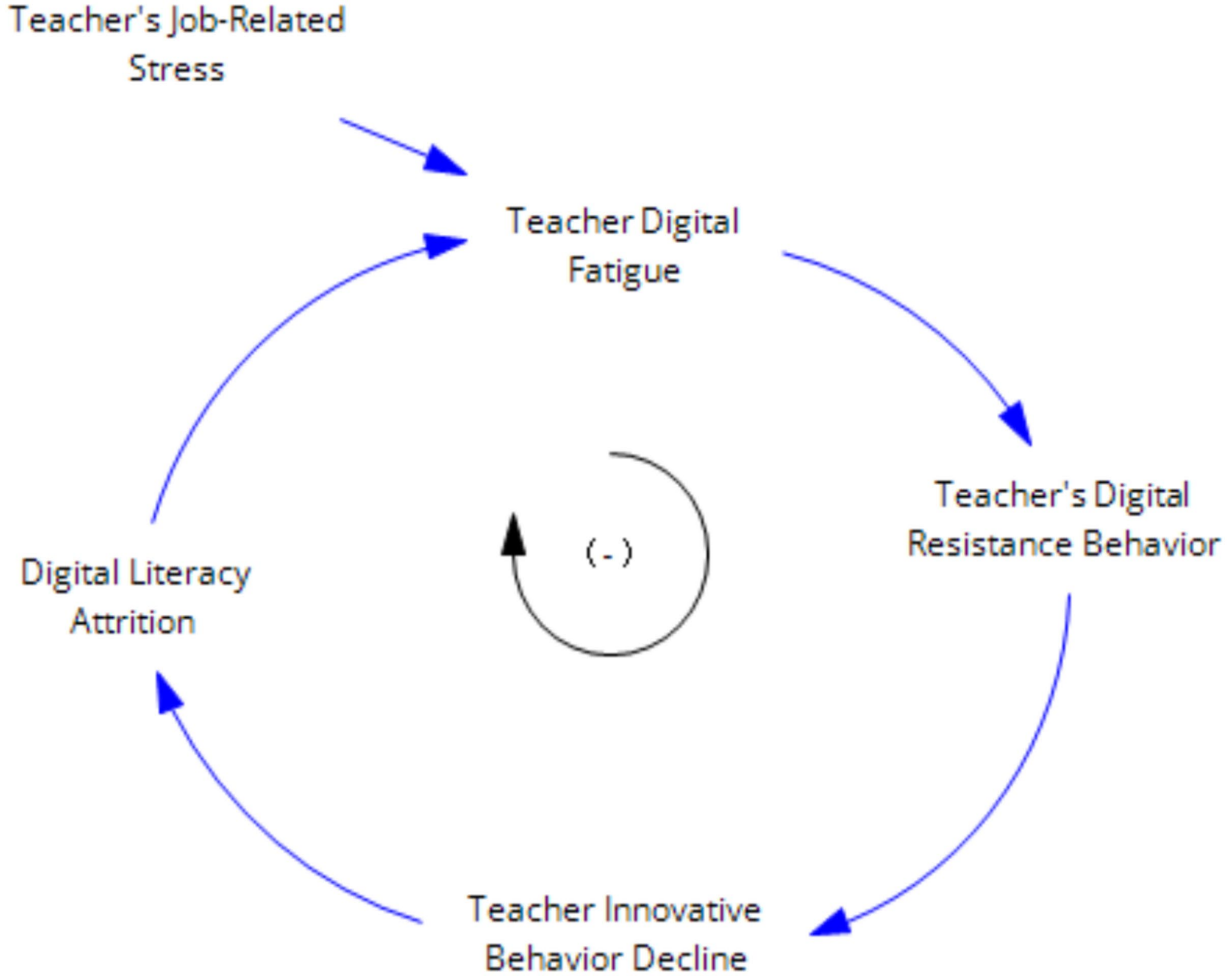
Teacher digital fatigue suppression feedback loop.

Teachers experience fatigue, burnout, and overload from continuous digital usage, leading to digital fatigue. This increases the likelihood of technical malfunctions and fosters self-doubt about their technology application behaviors, resulting in negative psychological effects and digital avoidance. Consequently, their innovative capabilities decline, and their original digital literacy deteriorates. As shown in Figure 7:

### 2.4 From theory to modeling: the applicability of system dynamics

The preceding discussion reveals that teachers’ digital literacy, psychological factors, and their innovative capabilities form a complex, multi-feedback dynamic system characterized by time delays and nonlinearity. Traditional linear statistical models alone cannot adequately elucidate their dynamic, cyclical causal relationships. System dynamics, however, serves as a fundamental tool for describing and understanding such complex nonlinear systems. Moreover, this approach can function as a “variable impact laboratory” for the system of transforming digital literacy into enhanced innovative capabilities. By adjusting parameters such as external environmental factors, it enables the prediction of variable responses under various conditions, thereby supporting evidence-based policymaking to promote teachers’ innovative behaviors. Consequently, this study adopts the system dynamics method, constructing an SD model to address the complexity outlined above, effectively simulate the system, and ensure the feasibility of the research.

Of course, there are other solutions to such problems, such as structural equation modeling and discrete event simulation. However, these methods mostly suffer from limitations like failing to capture time delays, difficulty in handling feedback, or mismatched modeling granularity. In contrast, the system dynamics modeling approach is best suited for addressing the nonlinear dynamic feedback system formed by teachers’ digital literacy, psychological factors, and their innovation capabilities. This aligns closely with the ultimate goal of this study—to explore the intrinsic mechanisms by which university teachers’ digital literacy influences their own innovation capacity.

In summary, this paper employs system dynamics modeling to explore the psychological drivers that stimulate the enhancement of university teachers’ innovation capabilities and clarify the mechanisms for improving teachers’ innovative abilities from a dynamic perspective.

### 2.5 Brief introduction to the research methods

System dynamics models can simulate the multiple feedbacks and nonlinearities existing in a system, making them suitable for theoretical construction research (Fisher, 2018). The SD model constructed in this study is a second-order model, also known as a model of a model (Awang et al., 2015), which is particularly applicable to simulation challenges where data are insufficient or variables are difficult to quantify. It is a modeling process of theoretical deduction (a model of theoretical narrative) (Denzin and Lincoln, 2018), emphasizing the rationality of theoretical deduction.

The validity of a second-order model depends on the theoretical logical reasoning process and the modeling process (Mansolf and Reise, 2017) rather than the data fitting degree of the model results. Thus, it can ensure the internal validity of theoretical modeling and system simulation. Therefore, this study selects a second-order model as the theoretical model for construction.

## 3 System modeling and simulation

### 3.1 Model construction

**Figure 8:**
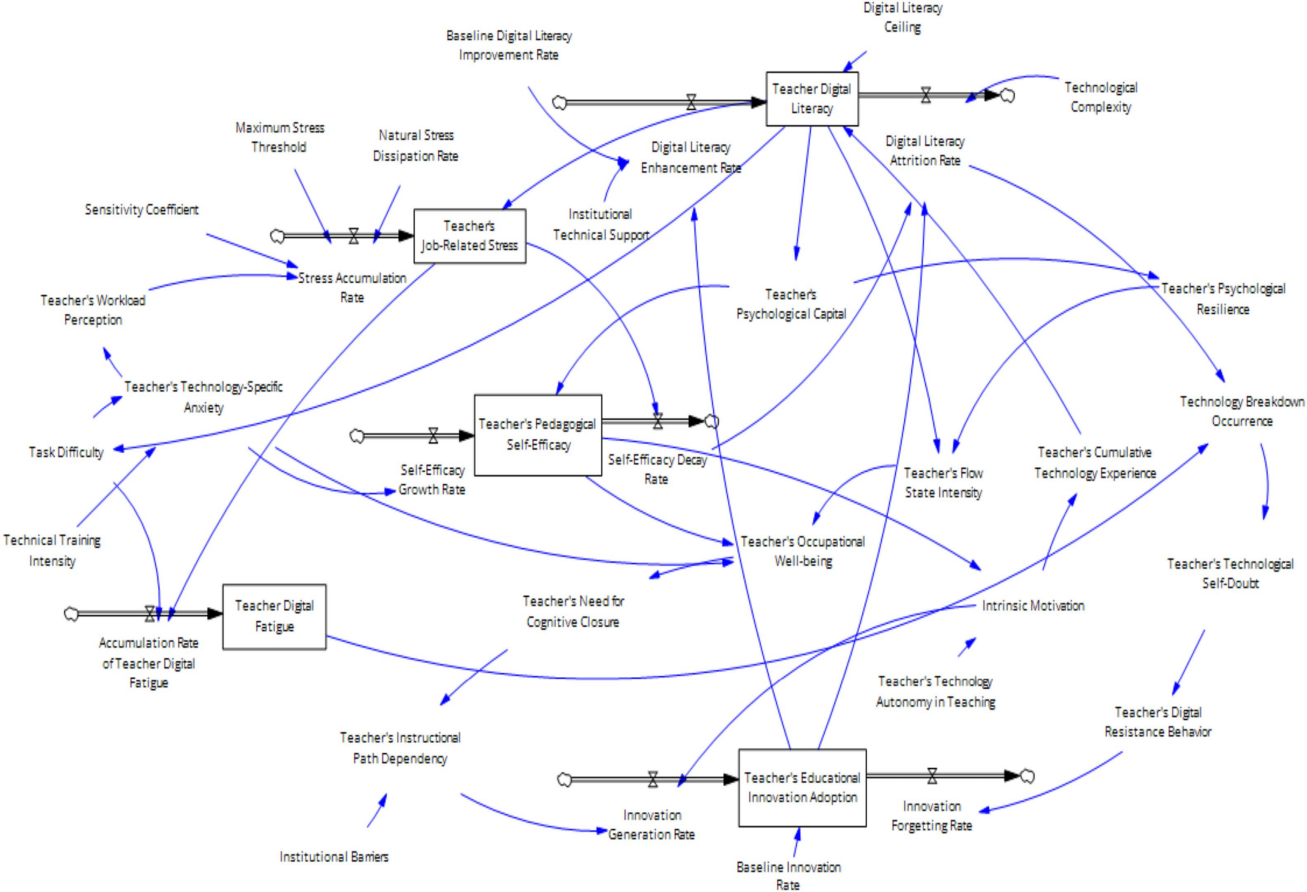
Teacher’s digital literacy stock and flow diagram.

The above five feedback loops jointly affect teachers’ digital literacy ability and the influence mechanism of teachers’ digital literacy on their own innovation ability. However, the causal feedback loop relationship reflects only the qualitative relationship and cannot express the quantitative relationship between various elements and the differences in variables of different natures. To clearly describe the relationships among the various elements of the system, this paper designs a system dynamics flow chart, as shown in Figure 6. This model contains three levels, namely, the digital ecology layer, the psychological capital layer and the innovation output layer (Figure 8).

Co-evolution process:

Step 1: Improving teachers’ digital literacy leads to more initial resources for individual teachers, enhances their ability to acquire further resources, promotes the accumulation of psychological capital, and increases their self-efficacy.

Step 2: Increasing teachers’ digital literacy reduces their technological anxiety. In the context of the digital transformation of education and the daily use of digital technology in the classroom, teachers’ workload is relatively reduced, thereby lowering the accumulation of work pressure and enhancing their self-efficacy in digital classrooms.

Step 3: The further accumulation of psychological resources, such as teachers’ self-efficacy, promotes a more positive mindset and work behavior among individual teachers, thereby enhancing their sense of happiness and intrinsic motivation.

Step 4: High digital literacy among teachers can reduce the occurrence rate of technical faults when using technology, decrease self-doubt behaviors, lower technological anxiety, and enhance technological self-efficacy, thereby further increasing teachers’ sense of happiness.

Step 5: The intensity of teachers’ flow experience and happiness affects their innovation behavior.

A: A high intensity of teachers’ flow experience enhances their sense of happiness during the teaching process, reduces their need for cognitive closure, decreases their reliance on traditional paths, stimulates innovative behaviors, increases the level of innovative behaviors, and enhances their own innovation capabilities.

B: A decrease in teachers’ happiness from using technology increases their need for cognitive closure, strengthens their reliance on traditional teaching paths, reduces the occurrence of innovative behaviors, and weakens their innovation capabilities.

Step 5: The effect of teachers’ innovation behavior level on their digital literacy.

A: An increase in teachers’ innovation behavior level increases their investment in the digital transformation of education, increases the complexity of the technology they face, increases their work pressure, increases their technological anxiety, and accelerates the forgetting of innovative behaviors, thereby to some extent inhibiting the level of teachers’ innovative behaviors.

B: An increase in teachers’ innovation behavior leads to breakthrough innovations, promotes the reinvestment of school resources, and further enhances teachers’ digital literacy.

### 3.2 Main parameters of the model and simulation methods

This section presents the internal structure of each subsystem according to the subsystem settings, including the main parameters of the model and the simulation equations.

#### 3.2.1 Main parameters

The 38 variables, variable names, properties and initial values contained in the three subsystems of this model are shown in Table 2.

**Table 2:** Model parameters and properties.

Level	Variable name	Properties	Initial value
Digital ecosystem layer	Teacher digital literacy	Stock	3.5
	Digital literacy enhancement rate	Flow	---
	Digital literacy attrition rate	Flow	---
	Baseline digital literacy improvement rate	Constant	0.02
	Digital literacy ceiling	Constant	90
	Institutional technical support	Constant	50
	Technological complexity	Constant	60
Psychological capital stratum	Teacher’s pedagogical self-efficacy	Stock	3
	Self-efficacy growth rate	Flow	---
	Self-efficacy decay rate	Flow	---
	Teacher’s job-related stress	Stock	4
	Stress accumulation rate	Flow	---
	Teacher’s psychological capital	Auxiliary	---
	Teacher’s flow state intensity	Auxiliary	---
	Teacher’s occupational wellbeing	Auxiliary	---
	Teacher’s technology-specific anxiety	Auxiliary	---
	Teacher’s need for cognitive closure	Auxiliary	---
	Teacher’s psychological resilience	Auxiliary	---
	Sensitivity coefficient	Constant	0.22
	Maximum stress threshold	Constant	100
	Natural stress dissipation rate	Constant	0.05
	Teacher’s workload perception	Auxiliary	---
	Task difficulty	Auxiliary	---
	Technical training intensity	Auxiliary	---
	Teacher’s technology autonomy in teaching	Auxiliary	---
	Intrinsic motivation	Auxiliary	---
	Teacher’s technological self-doubt	Auxiliary	---
	Teacher’s Digital Resistance Behavior	Auxiliary	---
	Teacher Digital Fatigue	Stock	---
	Accumulation Rate of Teacher Digital Fatigue	Flow	---
Innovation output layer	Teacher’s Educational Innovation Adoption	Stock	---
	Innovation Forgetting Rate	Flow	---
	Innovation Generation Rate	Flow	---
	Baseline Innovation Rate	Constant	0.02
	Institutional Barriers	Constant	2.5
	Teacher’s Cumulative Technology Experience	Auxiliary	---
	Teacher’s Instructional Path Dependency	Auxiliary	---
	Technology Breakdown Occurrence	Auxiliary	---

#### 3.2.2 Simulation equations

The important variable simulation equations involved in this study and their bases are shown in Table 3.

**Table 3:** Core variable simulation equations and their bases.

Variable name	The simulation equation formula of the core variables	Programming basis and description
Teacher digital literacy	=(Digital Literacy Enhancement Rate-Digital Literacy Attrition Rate)*Teacher Digital Literacy*(1-Teacher Digital Literacy/Digital Literacy Ceiling) + 0.5*Teacher’s Cumulative Technology Experience	(1) Standard logistic growth equation: r*x*(1-x/K) r = digital literacy_basic growth rate (0.02) x = digital literacy K = digital literacy_upper limit (100) (2) Technical Experience Influence Coefficient (0.5) – Technical experience has a positive incentive effect on teachers’ digital literacy
Digital literacy enhancement rate	=0.025*SMOOTH (Institutional Technical Support, 3) + Baseline Digital Literacy Improvement Rate+0.02*Teacher’s Educational Innovation Adoption	(1) School technical support includes teacher training, and in teacher training research, the “input-effect” conversion rate typically ranges between 20 and 30% [as noted by Desimone (2009) regarding the mediating role of training design quality]. This paper assumes a correlation coefficient of 0.25.
Digital literacy attrition rate	=0.05*(1 + Technological Complexity/4) + "Self-Efficacy Decay Rate”-0.02*Teacher’s Educational Innovation Adoption	(1) The decline in technical complexity and self-efficacy leads to the degeneration of digital literacy;
		(2) The impact coefficient of teachers’ innovative behavior level (0.02)---Teachers’ innovative behavior level can reduce the degeneration efficiency by approximately 2%
Teacher’s psychological capital	=SMOOTH (0.5*Teacher Digital Literacy, 3)	(1) According to the “resource gain” principle of the Conservation of Resources (COR) theory (Hobfoll, 1989) and Social Cognitive Theory (Bandura), psychological capital can accumulate through the acquisition of resources (in this study, the resource being digital literacy), with accumulation requiring time and experiencing conversion losses. Therefore, this study sets a delay period of 3 months and assigns an efficiency rate of 0.5 for the comprehensive conversion of digital literacy into psychological capital, indicating that external constraints and losses in the transformation of digital literacy remain at a relatively conservative and ordinary level.
Teacher’s pedagogical self-efficacy	=“Self-Efficacy Growth Rate”-"Self-Efficacy Decay Rate” + 0.3*Teacher’s Psychological Capital	(1) The standardized path coefficient of psychological capital on teachers’ self-efficacy ranges between 0.25 and 0.40 (Luthans et al., 2007). This study selects 0.3. (2) Initial value (3)----Based on the Likert scale (5-point), this study selects the general level of 3 points for teachers’ self-efficacy as the initial value.
Teacher’s flow state intensity	=0.5*Teacher Digital Literacy + IF THEN ELSE (Teacher Digital Literacy>4, Teacher’s Psychological Resilience*0.4, 0.2*Teacher’s Psychological Resilience)	(1) Teacher Digital Literacy Impact Coefficient (0.5) -- The correlation coefficient between teacher digital literacy and flow experience ranges between 0.4 ~ 0.6 (e.g., Coller and Scott, 2009), hence this paper adopts the value of 0.5;
		(2) Based on Fredrickson’s Broaden-and-Build Theory of Positive Emotions and the theory that psychological resilience can serve as a “buffer,” this study sets the teacher psychological resilience impact coefficient at 0.4 in high teacher digital literacy contexts and 0.2 in low digital literacy contexts.
Teacher’s occupational wellbeing	=0.25*Teacher’s Flow State Intensity+0.35*SMOOTH (“Teacher’s Pedagogical Self-Efficacy,” 4) -0.4*SMOOTH (“Teacher’s Technology-Specific Anxiety,” 2) + 3	(1) Teacher self-efficacy impact coefficient (0.35) ------ According to Self-Determination Theory, self-efficacy fulfills the “need for competence,” directly enhancing well-being; the standardized path coefficient of teacher self-efficacy on well-being typically ranges between 0.30 ~ 0.45 (e.g., Klassen and Chiu, 2010);
		(2) Teacher technology anxiety impact coefficient (0.4) ------ Technology anxiety has a relatively strong negative effect on teacher well-being (*β* ≈ −0.35 ~ −0.50), especially during periods of technological change;
		(3) The constant term represents baseline well-being
Teacher’s technology-specific anxiety	=0.6*IF THEN ELSE (Task Difficulty>3, Task Difficulty+0.5, 0) -0.3*SMOOTH (Technical Training Intensity, 3)	(1) Task difficulty influence coefficient (0.6) ------ Task difficulty (such as the complexity of new technologies) is a core driver of anxiety (Davis, 1989). The standardized path coefficient of teachers’ technological task difficulty on anxiety typically ranges from 0.5 to 0.7;
		(2) Using the IF THEN ELSE function, when task difficulty exceeds 3, an additional stimulating effect of 0.5 will be applied;
		(3) Technical training investment intensity coefficient (0.3) ----- Investment in technical training can reduce teachers’ technological anxiety by approximately 30%
Teacher’s need for cognitive closure	=4–0.4*"Teacher’s Occupational Well-being”	(1) High NFCC may hinder teaching innovation (such as rejecting new technologies) and reduce classroom flexibility, while teachers with high well-being are more likely to fulfill the needs for “autonomy” and “competence,” thereby tolerating ambiguity
Innovation forgetting rate	=0.7*Teacher’s Digital Resistance Behavior	(1) Digital Avoidance Behavior Impact Coefficient (0.7) - One unit of digital avoidance behavior will result in a 70% forgetting rate of innovative behavior.
Teacher’s educational innovation adoption	=MAX (0,(Baseline Innovation Rate-Innovation Forgetting Rate)* Teacher’s Educational Innovation Adoption+Innovation Generation Rate)	(1) The innovation behavior generation rate refers to the frequency at which teachers voluntarily attempt new methods, techniques, or strategies per unit time. Behavioral intention directly drives behavior generation (Ajzen, 1991; Ajzen, 2002), thereby influencing the level of innovative behavior.
		(2) MAX function—ensures that the level of teachers’ innovative behavior is >0.
		(3) Initial value = 2.5
Innovation generation rate	=IF THEN ELSE (Teacher’s Instructional Path Dependency>3, 0.05*Intrinsic Motivation, 0.03*Intrinsic Motivation)	(1) Teacher’s path dependency intensity influence coefficient (0.2)----This is the behavioral manifestation of NFCC (Need for Cognitive Closure), which reduces the generation of innovative behaviors by relying on historical choices to avoid the cognitive costs of re-evaluation.
		(2) Intrinsic motivation influence coefficient (0.03/0.05)-----A one-unit increase in intrinsic motivation leads to a 3% occurrence of teachers’ innovative behavior.
Teacher’s instructional path dependency	=0.5*Teacher’s Need for Cognitive Closure+0.3*Institutional Barriers	(1) Need for cognitive closure impact coefficient (0.5)-----Teachers’ need for cognitive closure leads to increased dependence on traditional paths;
		(2) Institutional barriers impact coefficient (0.3)----The inhibitory coefficient of institutional barriers on teacher innovation typically ranges from −0.15 to −0.25.
Stress accumulation rate	=Maximum Stress Threshold*(1-EXP (-Teacher’s Workload Perception*Sensitivity Coefficient)) -Natural Stress Dissipation Rate	(1) Sensitivity coefficient (0.2) – Set based on the 1996 Effort-Reward Imbalance theory. Individual sensitivity differences lead to varying rates of work stress accumulation.
		(2) For every unit increase in teacher workload, work pressure also rises;
		(3) Natural stress dissipation rate – The effect of natural reduction in stress levels is subtracted.
Teacher’s digital resistance behavior	=0.45*"Teacher’s Technological Self-Doubt”	(1) Teacher self-doubt behavior influence coefficient (0.45) ---- Teacher self-doubt behavior stimulates the occurrence of teacher digital avoidance behavior.

### 3.3 Model results and analysis

**Figure 9:**
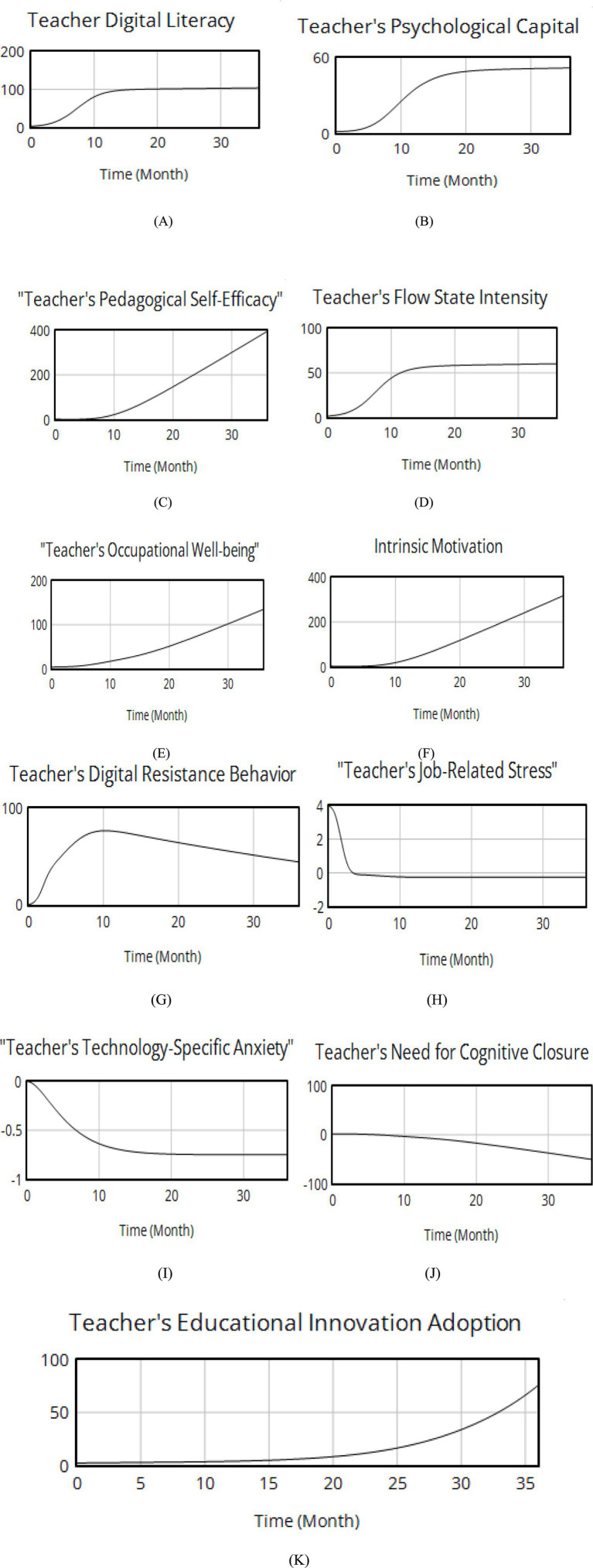
Basic operation results of the model.

According to the “resource gain” principle of Conservation of Resources Theory (COR, Hobfoll, 1989) and Social Cognitive Theory (Bandura, 2000a), psychological capital can accumulate through resource acquisition (digital literacy as the resource in this study). Accumulation requires time, and resource conversion entails loss. Therefore, this study inputs the aforementioned parameters and equations into the model, with a simulation period of 36 months and a time step of 0.0625. The basic simulation results are shown in Figure 9.

As shown in Figure 9A, the curve of teachers’ digital literacy first increases slowly, then accelerates at approximately 20 months, and finally slows naturally when it approaches the upper limit (Lucas et al., 2021). As shown in Figures 9B–F, with the improvement of digital literacy among college teachers, teachers’ psychological capital, self-efficacy, intensity of flow experience, happiness, and intrinsic motivation all show increasing trends. The accumulation of teachers’ psychological capital increases, self-efficacy is enhanced, and the intensity of flow experience and happiness also increases accordingly, reflecting a positive reinforcing relationship between teachers’ digital literacy and a series of psychological variables in this study. Psychological factors are the core drivers influencing teachers’ digital literacy, which aligns with the findings of Ning et al. (2025) and Wegerif (2024). As previously mentioned, this study uses the level of innovative behavior as a proxy for teachers’ innovative capacity. As shown in Figure 9I, the level of teachers’ innovative behavior also exhibits a growth trend under the influence of self-efficacy, flow experience intensity, and psychological capital, indicating the mediating role of psychological variables in this study. Teachers’ digital literacy indirectly stimulates their innovative behavior through elements such as teachers’ psychological capital, self-efficacy, flow experience intensity, well-being, and intrinsic motivation, which is also consistent with the conclusions of Mousavi and Ebrahimi (2024). Figure 9I reveals that as teachers’ digital literacy improves, their subjective perception of task difficulty weakens, and their technological anxiety shows a decreasing trend. Technological anxiety, as a negative psychological factor, limits teachers’ willingness to innovate by fostering resistance to using educational technology, thereby hindering the emergence of innovative behavior and suppressing teachers’ innovative capacity (Hopcan et al., 2024).

**Figure 10:**
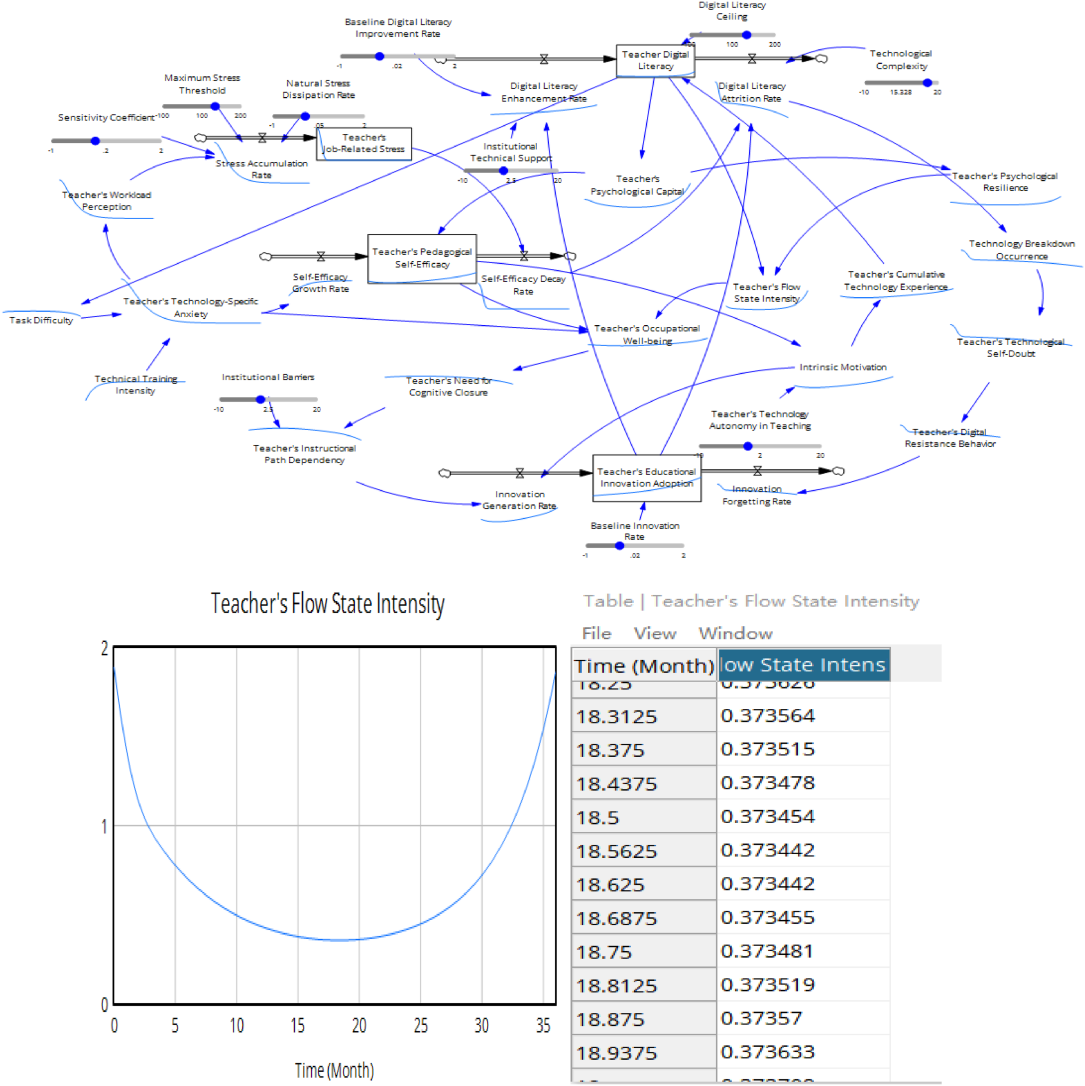
The running results of teachers’ flow experience intensity under changes in technical complexity.

Figure 10 shows the results of the intensity of teachers’ flow experience under changes in technical complexity. As shown in Figure 10, the research results reveal that when technical complexity is controlled for alone, the curve of teachers’ flow experience intensity significantly changes. Figure 9D indicates that under low technical complexity, as teachers’ digital literacy improves, the intensity of their flow experience increases monotonically and gradually flattens out in the later stage. However, Figure 10 shows that under extremely high technical complexity, as teachers’ digital literacy improves, the curve of their flow experience intensity takes on a “U” shape, showing a decreasing effect from 0--18 months and an increasing effect from 18 to 36 months, with a minimum point. It can be seen that when the cycle is approximately 0.5, under extremely high technical complexity, teachers’ flow experience intensity reaches its lowest point.

**Figure 11:**
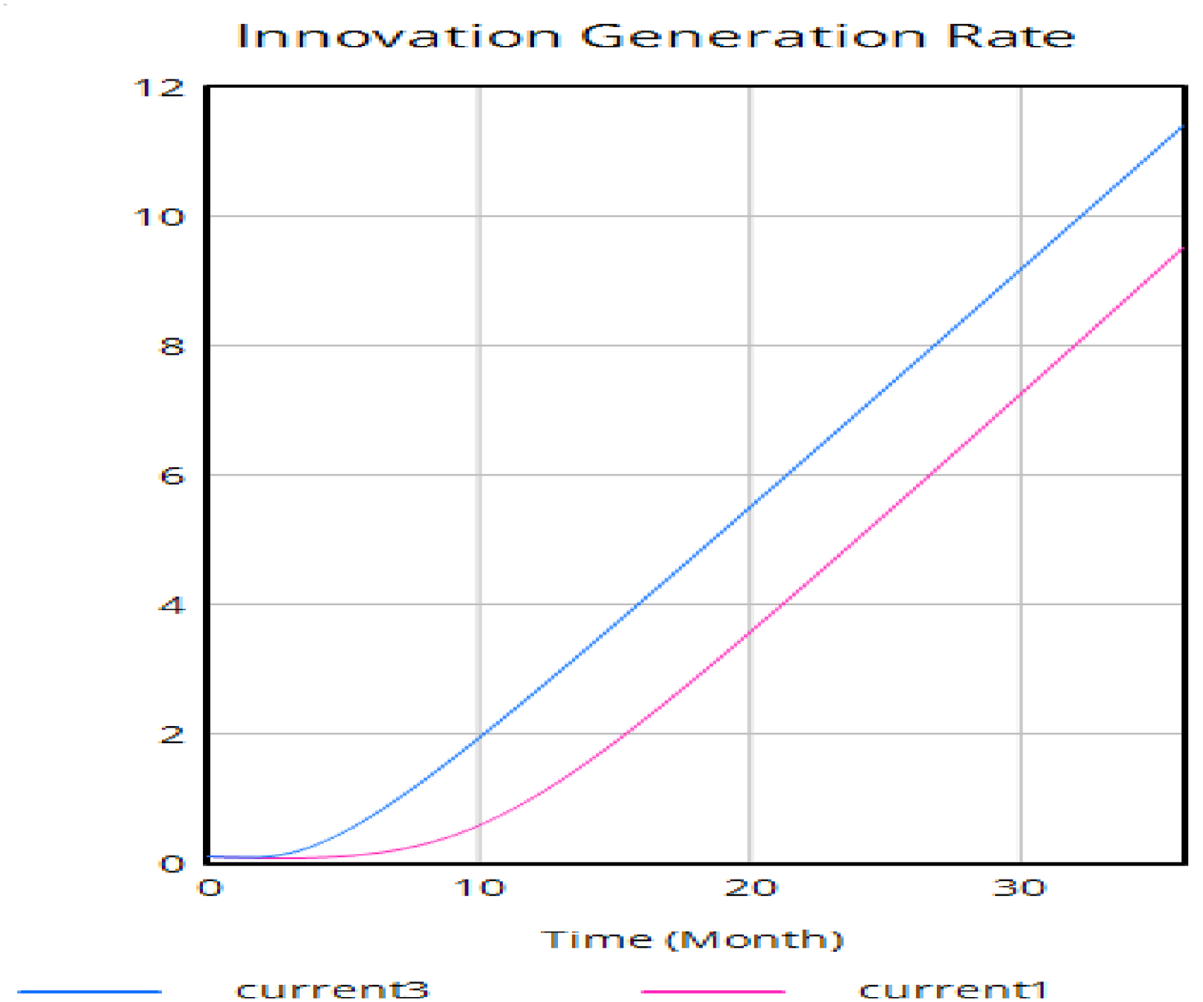
Comparison of the innovation behavior level curves of teachers.

This study used school support for technology—including teacher technology training, basic equipment support, and resource platforms—to control the variable of teachers’ digital literacy. Under otherwise unchanged conditions, the research compared the improvement rates of digital literacy between scenarios of low and high school technology support. The results are shown in Figure 11, where the blue curve represents the level of teachers’ innovative behavior under high school technology support, and the red curve represents the level under low school support. It can be observed that the curve of teachers’ innovative behavior is steeper when schools provide stronger technological support. The conclusion is that school technology support significantly impacts the improvement of teachers’ digital literacy—the stronger the support, the higher the teachers’ digital literacy. This aligns with the findings of Ertmer et al. (2012), Hopcan et al. (2024), Hsu et al. (2023), and Ning et al. (2025). School technology support serves as a crucial external condition for enhancing teachers’ digital literacy. Robust school support can significantly elevate teachers’ digital literacy, encourage greater integration of digital technology into classrooms, and strengthen teachers’ subjective willingness to use technology as well as their own innovative capabilities.

### 3.4 Sensitivity analysis of key variables

**Figure 12:**
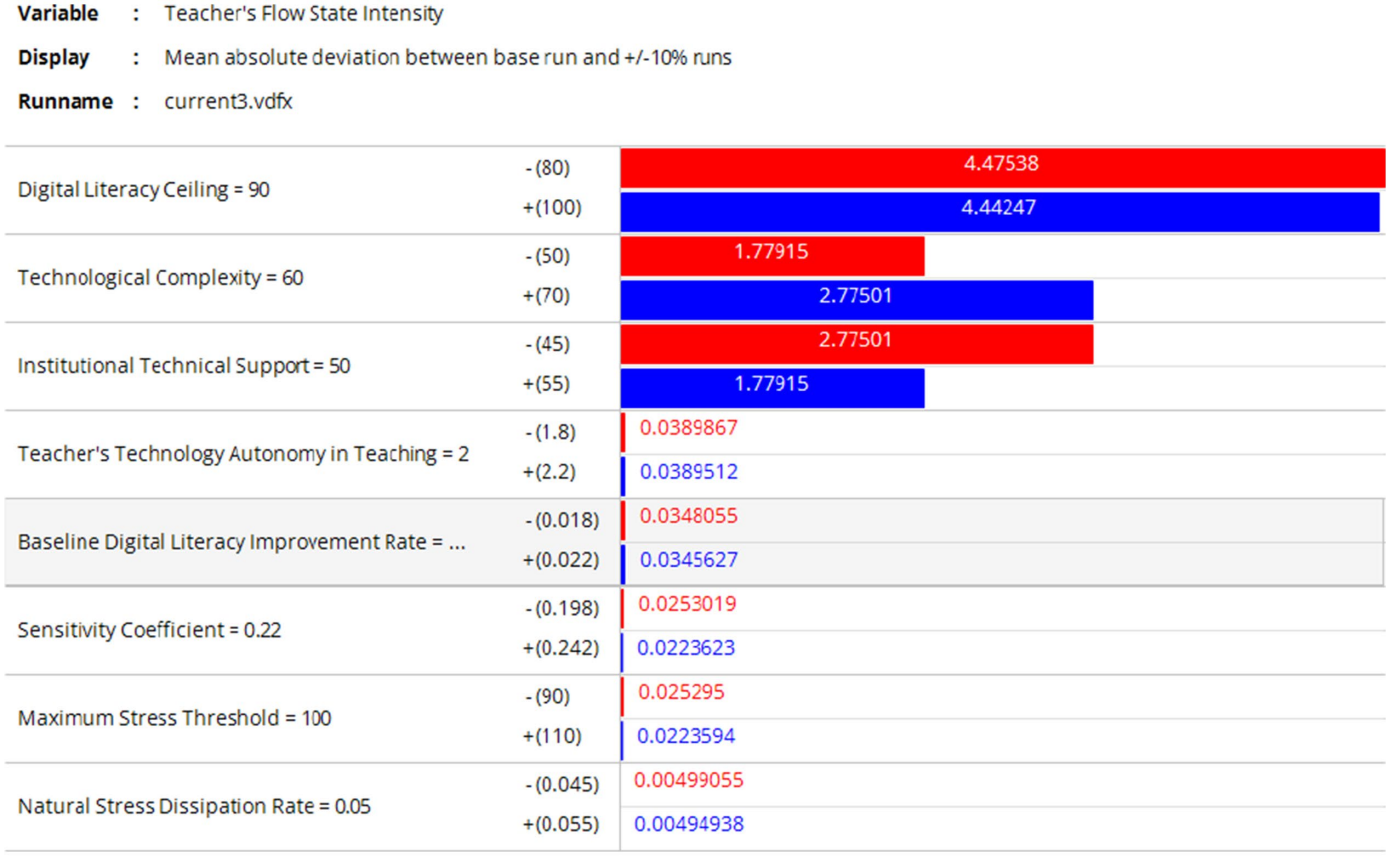
Sensitivity analysis results of multiple factors affecting the intensity of teachers’ flow experience.

To test the robustness of the proposed model, this study focuses on the intensity of teachers’ flow experience as a core variable linking the digital ecology layer and psychological capital layer. Conducting a sensitivity analysis over a 36-month simulation period, we examined the impact of multiple factors on teachers’ flow experience intensity. Under idealized assumptions, the baseline values for teachers’ digital literacy and technological complexity were set at 90 and 60, respectively, with school technical support at 50, while other factors remained at moderate ideal levels (see Figure 12). We analyzed the effects of ±10% variations in these factors on flow experience intensity. The results indicate that teachers’ digital literacy, technological complexity, and school technical support exert the most significant influence. As shown in Figure 12, reducing the baseline value of teachers’ digital literacy from 90 to 80 substantially weakened flow experience intensity, with an absolute deviation of 4.47538, confirming that lower digital literacy diminishes flow. Conversely, increasing digital literacy to 100 enhanced flow intensity, with a variation of 4.44247, demonstrating its reinforcing effect. When technological complexity decreased to 50, the mean absolute deviation was 1.77915, whereas an increase to 70 resulted in a deviation of 2.77501, indicating that higher complexity negatively impacts flow experience, while reduced complexity enhances it. School technical support also played a role: lowering the baseline from 50 to 45 yielded an effect value of 1.77915, whereas raising it to 55 resulted in 2.77501, suggesting that improved support fosters flow, while insufficient support weakens it. Other variables, such as teachers’ technological autonomy and maximum stress tolerance, had minor effects, ranging between 0.03 and 0.025. These findings align with the study’s earlier conclusions, reinforcing the proposed “digital literacy–flow experience feedback loop.”

**Figure 13:**
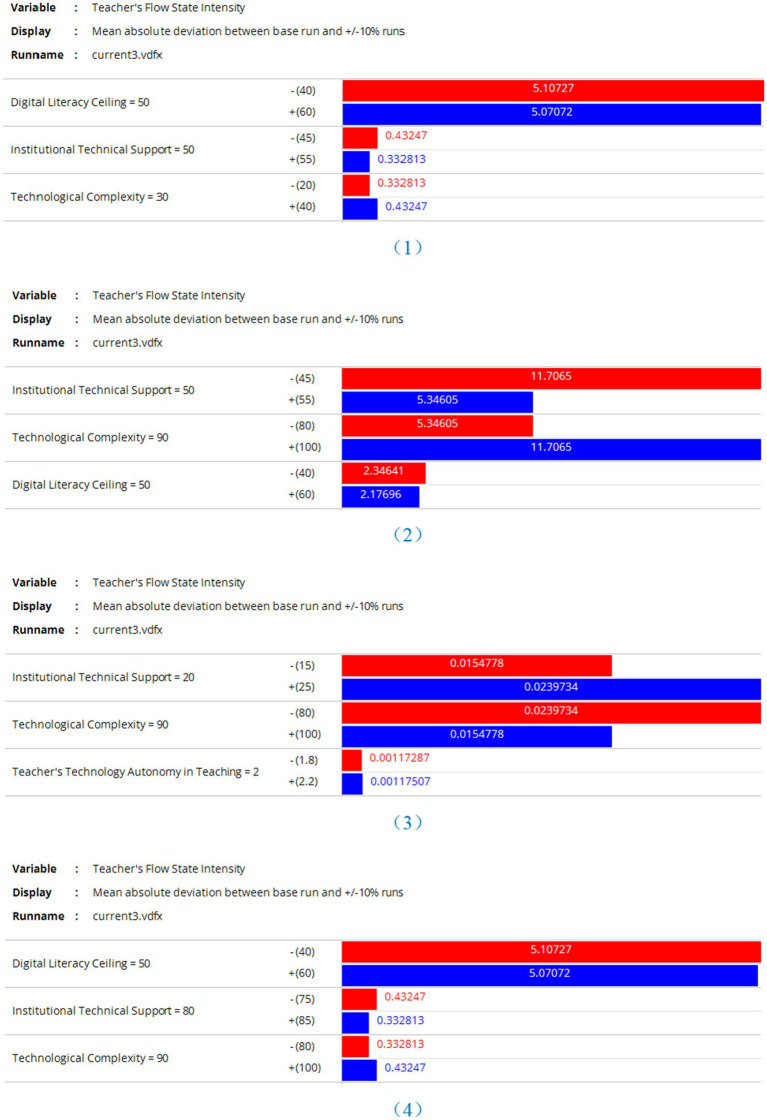
Sensitivity analysis results of key factors affecting the intensity of teachers’ flow experience under extreme values.

However, the model itself has certain limitations. To enhance the explanatory power of the analysis, this study conducts sensitivity analyses on multiple factors influencing the intensity of teachers’ flow experience under different scenarios. Four baseline settings are verified, as shown in Figure 13: (1) When technical complexity is extremely low, other variables remain unchanged; (2) When technical complexity is extremely high, other variables remain unchanged; (3) When school technical support is extremely low, other variables remain unchanged; (4) When school technical support is extremely high, other variables remain unchanged. The validation results show a high consistency with the aforementioned conclusions, indicating that when teachers’ digital literacy, technical complexity, and school technical support fluctuate within reasonable ranges, the variation logic, direction, and stage characteristics of teachers’ flow experience align with theoretical expectations—no logical contradictions or extreme-value-driven scenarios occur, demonstrating the model’s strong structural robustness.

## 4 Conclusion and implications

### 4.1 Conclusion

As mentioned earlier, this study follows the dominant logic of the “digital literacy of university teachers—various influencing factors (black box)—their own innovation ability” to explore the “black box mechanism” and raises the question of how variables such as teachers’ psychological capital, intensity of flow experience, self-efficacy, happiness, and technology anxiety act as mediating variables in the influence mechanism. This paper constructs an SD model to incorporate teachers’ digital elements, psychological capital, self-efficacy and happiness, as well as innovation ability, into the same system. By inputting the main parameters and simulation equations for simulation through a short-term analysis of 36 months, the main results of this study are as follows:

First, teachers’ digital literacy drives the improvement of their own innovation capability through the chain transmission of psychological variables. The study finds that teachers’ digital literacy affects their innovation capability, but this influence is not direct. Instead, it operates indirectly through a series of psychological mediating variables such as teachers’ self-efficacy, flow experience intensity, and well-being. When teachers’ digital literacy improves, their psychological capital, self-efficacy, and flow experience intensity are enhanced, stimulating their subjective willingness to absorb and learn digital technologies and strengthening their intrinsic motivation to integrate digital technologies into the classroom. This encourages teachers to spontaneously explore new approaches in digital teaching and classroom design, ultimately fostering the emergence of innovative behaviors and enhancing their innovation capability.

Second, the level of teachers’ digital literacy influences their subjective perception of task difficulty. The study finds that improving teachers’ digital literacy can reduce their subjective perception of task difficulty, thereby alleviating their technological anxiety, lowering the work pressure associated with using technology in teaching, and boosting their self-efficacy and well-being. This, in turn, enhances intrinsic motivation, promoting the generation of innovative behaviors and elevating the level of teachers’ innovative actions. Enhancing teachers’ digital literacy can create a favorable psychological environment for the leap in their innovation capability, forming a virtuous cycle of “improved digital literacy—reduced technological anxiety—activated intrinsic motivation—output of innovative behaviors.”

Third, the study also finds that institutional support for technology and technological complexity are significant external factors in how teachers’ digital literacy affects their innovation capability, playing a notable role in the mechanism by which digital literacy influences the level of teachers’ innovative behaviors. On one hand, institutional technical support (e.g., technical training, hardware facility support, provision of digital technology resources) can effectively help teachers enhance their digital literacy, reduce their perceived difficulty with digital technologies, strengthen their intrinsic motivation, and promote the generation of innovative behaviors. On the other hand, technological complexity can directly affect the barriers teachers face in applying digital technologies. Lower technological complexity can reduce work pressure and avoidance behaviors, stimulating the emergence of innovative behaviors. Together, these factors provide strong external support for the impact of teachers’ digital literacy on their innovation capability.

Fourth, the “U”-shaped curve of flow experience under extreme technological complexity. The study found that when technological complexity reaches its maximum, the intensity curve of teachers’ flow experience no longer increases monotonically. As teachers’ digital literacy improves, the intensity curve of their flow experience exhibits a “U” shape, showing a decreasing effect from approximately 0 to 18 months and an increasing effect from 18 to 36 months, with a lowest point existing.

Fifth, the degradation of teachers’ digital literacy triggers a vicious cycle of digital avoidance and decline in innovation capability. The accelerated degradation of teachers’ digital literacy increases the likelihood of technical failures during use, exacerbates self-doubt behaviors when employing technology, and consequently leads to more instances of digital avoidance and stronger forgetting of innovative practices, further diminishing teachers’ level of innovative behavior. Moreover, as the level of innovative behavior declines, it inversely impacts the rate of digital literacy degradation, accelerating the deterioration of teachers’ digital literacy and thus forming a vicious cycle. Second, a high level of digital literacy has an impact on the subjective task difficulty of teachers. The improvement of teachers’ digital literacy leads to a reduction in subjective task difficulty, a decrease in teachers’ technology anxiety, a reduction in the work pressure of teachers using technology in teaching, an increase in teachers’ self-efficacy and happiness, and then an increase in intrinsic motivation, promoting teachers’ innovative behavior and improving the level of teachers’ innovative behavior.

Sixth, teachers’ digital fatigue plays a suppressive role in the process where digital literacy affects their innovative capabilities. After prolonged application of digital technologies in teaching, educators experience exhaustion and overload, leading to digital fatigue. This increases the likelihood of technical failures, fosters self-doubt regarding technology use, and prompts digital avoidance behaviors. Ultimately, these factors reduce innovative behaviors among teachers, resulting in a significant decline in their level of innovative practices.

### 4.2 Theoretical contributions

In response to the insufficient research efforts in the literature on the impact of the “digital literacy of university teachers on their own innovation ability” and the fact that most of the research has focused on static empirical analysis with few dynamic perspective studies and has been based on the system dynamics method, this paper constructs a system dynamics model from the digital ecological layer to the psychological capital layer and then to the innovation output layer, uses the system dynamics method to describe and quantify the relationships among teachers’ digital literacy, psychological capital and other psychological factors and teachers’ innovation ability, simulates the process of the impact of teachers’ digital literacy on their own innovation ability, and identifies the influence mechanism of teachers’ digital literacy on their own innovation ability. The following theoretical contributions are made:

First, exploring the impact mechanism of teachers’ digital literacy on their own innovation capabilities contributes to the field of educational modernization and digital transformation. While existing cutting-edge research has acknowledged the importance and timeliness of digital literacy, most studies remain theoretical and seldom focus on clarifying the underlying mechanisms. For example, Javier TOURÓN et al. highlighted in their research that the integrated use of technology in classrooms can instantly provide information and improve teaching practices (Tourón et al., 2018). Artificial intelligence, as the core engine of educational digital transformation, demonstrates transformative potential in the rapidly evolving educational landscape (Karataş and Yüce, 2024; Celik, 2023; Ning et al., 2025). AI-driven education can advance learning practices, support teaching, and create more personalized learning opportunities for students (Mustafa et al., 2024). Future teachers, as digital natives who use technology in daily life, can greatly benefit from implementing these applications in their teaching processes (Guillén-Gámez et al., 2020). This study, however, unravels the black box issue governed by the logic of “university teachers’ digital literacy—various influencing factors (black box)—personal innovation capabilities,” revealing the impact mechanism of teachers’ digital literacy on their innovation abilities. It constructs a dynamic system model spanning the digital ecological layer, psychological capital layer, and innovation output layer. By examining the dynamic interrelations across these stages, the research not only transcends the limitations of traditional static theories regarding stage interactions but also deepens the application of dynamic system theory in educational transformation.

Second, this study revealed the promoting role of psychological mediating variables such as psychological capital, teachers’ self-efficacy, teachers’ happiness, and intrinsic motivation in the output of teachers’ innovative behaviors, which can stimulate improvements in teachers’ innovative ability. This finding corresponds to the analysis of social cognitive theory, resource conservation theory, and self-determination theory. As the digital literacy of teachers serves as an initial resource and self-efficacy serves as a psychological resource, individuals with abundant initial resources have stronger resource acquisition capabilities and can exhibit more positive attitudes and work behaviors. Therefore, by enhancing teachers’ digital literacy, psychological mediating variables such as teachers’ self-efficacy and the intensity of their flow experience will also be strengthened, stimulating the occurrence of teachers’ innovative behaviors and promoting the improvement of their innovative ability. This aligns with the research of Spiteri and Chang Rundgren, indicating that teachers’ digital literacy’s impact on innovation capability can be studied through the pathway of willingness to innovate (Spiteri and Chang Rundgren, 2020). The findings of this study are an expansion of social cognitive theory, resource conservation theory, and self-determination theory in the context of modern educational digital transformation, enriching these theories and having contemporary significance.

### 4.3 Practical implications

First, it provides insights for relevant policy formulation, aiding in the rational construction and optimization of a digital literacy training system tailored to the real-world work scenarios of university faculty. This strengthens the implementation of institutional support for teachers in using technology during teaching and leveraging it more effectively. Against the backdrop of the digital era and global digital transformation, artificial intelligence exerts profound impacts on educational models, making the cultivation of university teachers’ digital literacy highly significant in practice. Public sectors and higher education institutions across nations should prioritize this and adopt a series of measures to enhance teachers’ digital literacy. Examples include government-led systematic training programs such as “Technology in the Classroom,” spearheaded by universities to elevate and broaden teachers’ intrinsic digital knowledge base; breaking down data silos to improve data-sharing capabilities; and increasing university investments in digital equipment to fully realize the application value of modern smart facilities, thereby providing faculty with better platforms for integrating technology into teaching.

Second, it facilitates the construction of an integrated knowledge framework where pedagogical knowledge, technological knowledge, and subject knowledge are deeply intertwined. This enables teachers to better couple knowledge from different domains during digital teaching practices, reducing their subjective perception of technological complexity and alleviating technological anxiety. Consequently, this lowers teachers’ cognitive closure needs and fosters higher intrinsic motivation and autonomy for innovative behaviors. For instance, the emergence of the TPACK framework emphasizes not the development of expertise in individual technologies but a mindset that helps teachers plan effective technology integration across technical, pedagogical, and content areas (Dalton, 2012; Karataş and Yüce, 2024; Bećirović, 2023), promoting optimized and innovative teaching. It also encourages the development of new skills, such as applying constructivist methods to teaching, learning, and lesson design. Teachers assume diverse roles and systematically organize varied activities using technology in response to student needs (Wake and Whittingham, 2013).

Third, it offers an institutional support-based approach to mitigate the stress and anxiety induced by technology and avoidance behavior. Research indicates that the core triggers of teachers’ technological stress, digital fatigue, and digital avoidance lie in the mismatch between their digital literacy and demands, as well as their subjective perception of digital technology complexity. Accordingly, universities can establish “technology suitability assessment techniques” to introduce digital teaching tools as needed or conduct periodic digital technology training. This reduces teachers’ subjective perception of technological complexity and prevents them from falling into the vicious cycle of “digital technology stress → digital fatigue → digital avoidance → diminished innovative capacity.”

Lastly, it provides a reasonable developmental pathway for educators by enhancing attention to the psychological well-being of university faculty, focusing on improving their flow experience, self-efficacy, and sense of fulfillment when using digital technologies. Public sectors should deeply understand and closely monitor the psychological dynamics of university teachers, fostering a correct and positive attitude toward improving their digital literacy and innovative behaviors. This strengthens faculty confidence in enhancing their innovative capabilities, making them more willing to actively embrace higher digital literacy and practice digital innovation. Efforts should also be made to cultivate a supportive psychological environment and an atmosphere conducive to autonomous improvement in digital literacy among university faculty.

### 4.4 Research gaps and future directions

Certainly, this study has certain limitations that warrant further exploration in subsequent research. First, the coefficients of influence on variables such as university teachers’ flow experience and self-efficacy were not obtained through independent sampling or data coding procedures but were instead summarized and refined based on existing authoritative studies, lacking more standardized and comprehensive data on influence coefficients. Second, system dynamics inherently relies on self-assumed data, while existing research designs seldom address the dynamic mechanisms of digital literacy and creativity, nor do they adequately explain the relationships between psychological variables. As a result, the quantitative process has shortcomings, and the modeling approach appears somewhat arbitrary. Third, this study only identified two inhibitory loops, which significantly deviates from the reality of improving teachers’ digital literacy, reflecting an idealized scenario. Moreover, the dynamic interactive effects of external variables were not fully considered. Fourth, longitudinal empirical validation is lacking, and the universality of core patterns as well as the practical applicability of the model require further investigation. Therefore, future research could include longitudinal empirical tests, expand variable dimensions and interaction analyses, and explore the relationships among teachers’ digital literacy, psychological capital, and the leap in their own innovation capabilities.

## Data Availability

The raw data supporting the conclusions of this article will be made available by the authors, without undue reservation.
